# The effect of dilated cardiomyopathy with moyamoya disease in a 31-year-old Chinese man: A case report

**DOI:** 10.1097/MD.0000000000031675

**Published:** 2022-12-16

**Authors:** Xia Yun Dong, Jie Yang, Chuan Hua Yang

**Affiliations:** a Shandong University of Traditional Chinese Medicine, Jinan, China; b Department of cardiovascular medicine, Affiliated Hospital of Shandong University of Traditional Chinese Medicine, Jinan, China.

**Keywords:** case report, combination of Chinese traditional and Western medicine, dilated cardiomyopathy, moyamoya disease, young patient

## Abstract

Dilated cardiomyopathy (DCM) is a clinically common and refractory disease; however, few cases of dilated cardiomyopathy have been reported in patients with moyamoya diseases treated by combining traditional Chinese Medicine (TCM) and Western medicine, which has a higher risk of rehabilitation.

**Case Summary:**

A 31-year-old man was admitted due to paroxysmal chest tightness and shortness of breath. He denied a history of DCM, hypertension, diabetes, pericarditis, smoking, and alcohol consumption. On admission, his transesophageal echocardiography (Fig. 1A) showed the larger heart with poor myocardial systolic function (left ventricular end diastolic diameter [LVEDd] 60 mm, left ventricular ejection fraction [LVEF] 38% [Teich]). On day 14 of admission, heart-related indicators were better than before.

**Conclusion:**

The present case is the first report demonstrating appearance the dilated cardiomyopathy (DCM) and moyamoya disease simultaneously in a 31-year-old Chinese man, aimed to report the treatment of such patients using a combination of TCM and Western medicine and analyzing the necessity and advantages of using this treatment for patients suffering from DCM and moyamoya disease, so as to improve the level of clinical diagnosis and treatment of such diseases.

**Figure 1.**
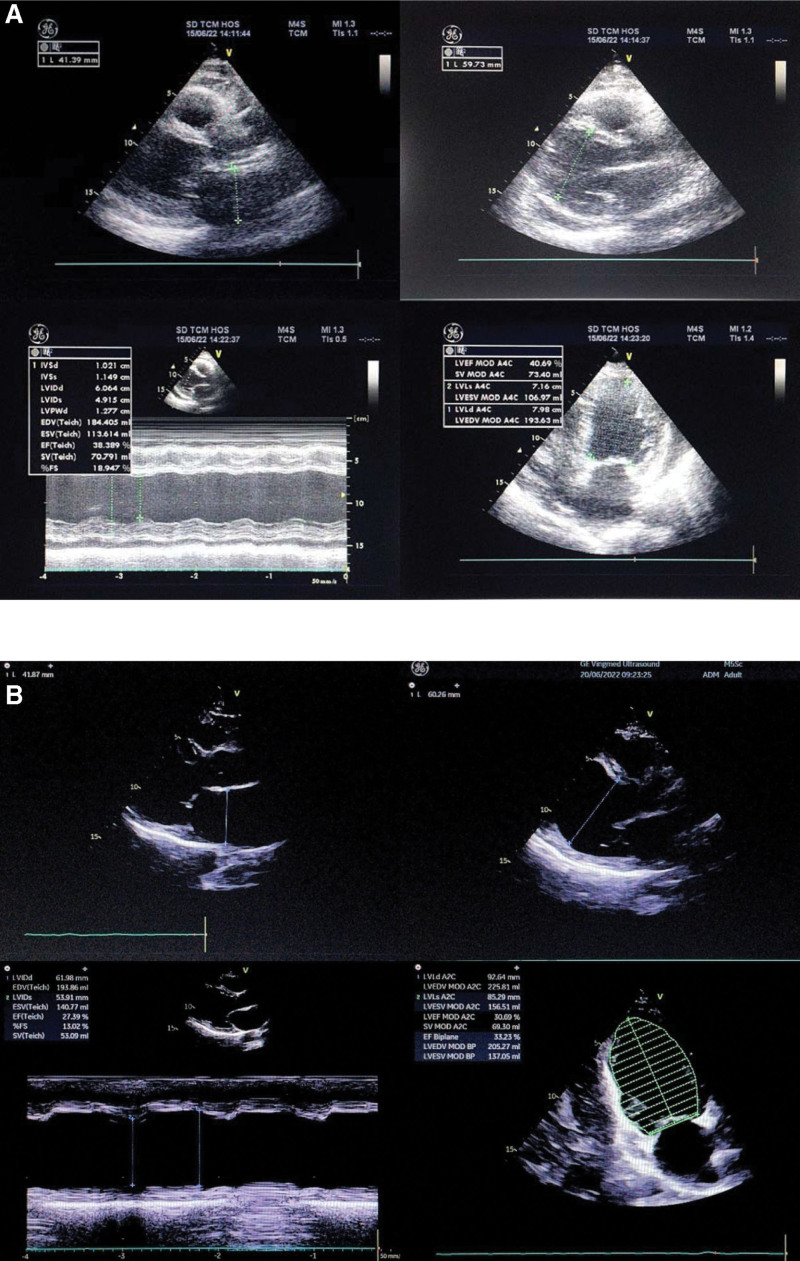
(A) Cardiac ultrasound index during hospitalization. (a) Cardiac ultrasound on June 15, 2022: ultrasonic findings of dilated cardiomyopathy (LAED 41 mm LVEDd 60 mm); mitral regurgitation (moderate); tricuspid regurgitation (moderate); pulmonary hypertension (mild to moderate); reduced left ventricular systolic and diastolic function (LVEF 38%, FS 19%); and pericardial effusion (small amount). (B) Cardiac ultrasound on June 20, 2022: myocardial lesions; left heart enlargement (LAED 42 mm, LVED 60 mm); mitral regurgitation (mild to moderate); tricuspid regurgitation (mild); pulmonary hypertension (mild); reduced left ventricular systolic function (LVEF 27%, FS 13%); and pericardial effusion (small amount). FS = fractional shortening, LAED = left atrium end diastolic, LVED = left ventricular end diastolic diameter, LVEF = left ventricular ejection fraction.

## 1. Introduction

Dilated cardiomyopathy (DCM)^[1–3]^ is a kind of heterogeneous cardiomyopathy mainly characterized by reduced myocardial systolic function and ventricular enlargement. Its clinical manifestations are a gradually enlarged heart, reduced ventricular systolic function, heart failure, ventricular and supraventricular arrhythmias, conduction abnormalities, thromboembolism, and sudden death. Echocardiography is an important method for diagnosing and evaluating DCM. In terms of Western medicine, many researches and applications in recent years include β-blockers, angiotensin-converting enzyme inhibitors/angiotensin receptor blockers/angiotesin receptor-neprilysin inhibitors (angiotensin-converting enzyme inhibitors/angiotensin receptor blockers/angiotes in receptor-neprilysin inhibitors), aldosterone receptor antagonist, dopamine, milrinone, levosimendan, and other drugs that increase positive myocardial contractile force. Traditional Chinese Medicine (TCM) believes that DCM is closely related to the heart. It also has to do with the liver, spleen, lung, and kidney. The pathogenesis of this disease is mainly characterized by asthenia in origin and asthenia in superficiality. Asthenia in origin refers to asthenia of yang in the heart and asthenia of qi and yin. Asthenia in superficiality refers to the stagnation of qi, blood stasis, phlegm, dampness, cold coagulation, and so forth, and even mixed with a combination of asthenia and sthenia, leading to the heart vessel blockage stasis and the disease. Therefore, the clinical treatment principle is to treat asthenia and reduce sthenia. Moyamoya disease,^[4]^ as a unique cerebrovascular disease, is mainly characterized by chronic stenosis of bilateral internal carotid arteries and the formation of an abnormal vascular network called moyamoya vessels. The gold standard for the diagnosis of moyamoya disease is stenosis or occlusion at the distal end of the internal carotid artery and/or anterior cerebral artery and/or initial segment of the middle cerebral artery and the presence of an abnormal vascular network at the base of the skull in the arterial phase found by digital subtraction angiography,^[5]^ which helps evaluate collateral circulation from the perspective of bleeding. Magnetic resonance imaging and magnetic resonance angiography can be used as alternatives to routine examinations. In Western medicine, revascularization is the main treatment for moyamoya disease; drugs are only applicable to symptomatic supportive treatment or perioperative management.^[6]^ TCM believes that the clinical symptoms of moyamoya disease are similar to those of “stroke.”^[7]^ In recent years, Kim et al, through epidemiological investigation and study, pointed out that in east Asia or Europe and the United States, the prevalence and incidence of moyamoya disease increased every year.^[8]^ With the increase in the number of onset and development of treatment theory, the advantage of combining TCM and Western medicine treatment of various diseases and prognoses has gradually been highlighted. The combination of TCM and Western medicine can reduce the mortality caused by DCM and moyamoya disease, as well as hospitalization rates, and improve the quality of life of patients. A case of DCM complicated with moyamoya disease treated by integrated TCM and Western medicine in Affiliated Hospital of the Shandong University of TCM was reported as follows to improve the understanding of this disease.

## 2. Case presentation

A 31-year-old male patient was admitted to our hospital due to “paroxysmal chest tightness and shortness of breath for >4 days.” The patient’s family members said that the patient had paroxysmal chest tightness, accompanied by palpitation, shortness of breath, and fatigue after the activity 4 days ago, and did not receive systematic treatment. Then, the aforementioned symptoms recurred and became worse after activities, with mild chest pain lasting for about 3 to 5 min. He was admitted to the outpatient department of our hospital. We performed some lab tests on the indicators: cardiac troponin T: 25.11 pg/mL. The patient was admitted to our hospital for further specialized treatment. At admission, he suffered from paroxysms of chest tightness and shortness of breath, occasional palpitation and chest pain, no shoulder and back radioactive pain, fatigue, occasional dizziness, headache, abdominal pain, and distension, nausea, and vomiting. He had no abnormal symptoms in eating, sleeping, defecation, and urination. The patient denied a history of hypertension, diabetes, pericarditis, smoking, and alcohol consumption.

The routine physical examination after admission revealed the following: temperature 36.5℃, pulse 93 times/min, respiration 17 times/min, blood pressure 156/123 mm Hg (R), and 147/115 mm Hg (L). The physical examination of the medical system showed a normal chest, coarse respiratory sounds in both lungs, a small amount of wet rales at the bottom of the lungs, no pleural frictional sound, rhythm, low and blunt heart sound, and mild edema in both lower limbs. The physical examination of the nervous system showed that the patient was in a poor mental state, with no obvious abnormalities.

After admission, the patient improved the relevant auxiliary examination: international standard ratio: 1.39; prothrombin time: 16.3 second; D dimer: 1.62 µg/mL; C-reactive protein (CRP): 12.9 mg/L; N-terminal pro brain natriuretic peptide: 3517 pg/mL; cardiac troponin T: 21.56 pg/mL; and white blood cell count (WBC): 11.08 × 10^9/L. The WBC reexamination on alternate days revealed the following: 9.25 × 10^9/L; transesophageal echocardiography (Fig. 1B): the ultrasonic findings of DCM (left atrium end diastolic 42 mm; left ventricular end diastolic diameter [LVEDd] 60 mm); mitral regurgitation (moderate); tricuspid regurgitation (moderate); pulmonary hypertension (mild to moderate); reduced left ventricular systolic and diastolic function (left ventricular ejection fraction [LVEF] 27% and fractional shortening 13%); and pericardial effusion (small amount). Magnetic resonance imaging of the brain revealed the following: The right cerebellar pontine wall and cerebellar hemisphere acute infarction possible; the left parietal lobe softened foci; manifestations of multiple cerebral ischemic infarcts; bilateral anterior cerebral artery, middle cerebral artery, and bilateral posterior cerebral artery occlusion, more localized stenosis in the left internal carotid artery intracranial segment and right internal carotid artery siphon segment, as well as local occlusion of the left internal carotid artery; abnormal changes in brain blood vessels, which were considered as magnetic resonance angiography manifestations of moyamoya disease.

TCM diagnosis showed the following: heart failure, qi deficiency, and blood stasis, cardiac distension, ischemic stroke, and megrim. Western medicine diagnosis included the following: acute heart failure, DCM, acute cerebral infarction, and hypertensive emergency. The onset of the patient was sudden and reached a peak immediately after admission, which was consistent with the symptoms of acute heart failure and acute cerebral infarction. The patient denied a history of pericarditis. Although the WBC increased slightly on examination at admission, it returned to normal the next day; therefore, the possibility of acute pericarditis was ruled out. The patient denied a history of hypertension, and cardiac ultrasound indicated that the interventricular septum was 10 mm. Therefore, it was disregarded that acute heart failure and DCM were caused by hypertension. Considering that the patient was at a low risk of arteriosclerotic cardiovascular disease with few arteriosclerotic cardiovascular disease risk factors, which was not enough to cause severe coronary atherosclerotic venereal disease, it was speculated that acute heart failure was induced by DCM and acute cerebral infarction was caused by moyamoya disease. According to the DCM staging of multi-center clinical trial data in China,^[9]^ this patient belonged to the middle stage of DCM (heart function grade II–III, with presenting symptoms such as extreme fatigue, labial dyspnea, and palpitation; LVEDd > 60–70 mm; LVEF 30%–40%). The treatment included sacubitril + valsartan sodium tablets to inhibit myocardial remodeling, bumetanide, and torsemide to improve heart failure, metoprolol succinate sustained-release and ivabradine hydrochloride to control the ventricular rate, and intravenous pumping of isosorbide dinitrate with recombined human brain natriuretic peptide to dilate the coronary artery. Patients with heart failure needed diuretics to reduce cardiac preload and correct heart failure. However, diuretics could increase the level of uric acid in the body, thus aggravating hyperuricemia. Studies^[10]^ showed that hyperuricemia not only aggravated gout but also was an important risk factor for acute cerebral infarction. The blood test results of the patient after admission showed the following: uric acid tendency was 968 mmol/L, which suggested high–uric acid hematic disease. The treatment of blood uric acid was required for patients with acute cerebral infarction.^[11,12]^ Also, the application of diuretics in patients with heart failure was essential. Therefore, the torasemide dose was changed from 40 mg to 20 mg, and combined with the oral drug sodium bicarbonate to correct high–uric acid hematic disease. Dynamic monitoring was needed in patients with high blood uric acid levels. Although the patient in the present case was in the acute stage of cerebral infarction, considering the patient’s international standard ratio as 1.29 and prothrombin time 16.3 seconds, the thromboelastogram indicated that the coagulation factor function was low. Patients with acute cerebral infarction are treated with low–molecular weight heparin within 24 hours, and the bleeding risk is higher. Therefore, thrombolysis was not considered temporarily in the present case, and drugs were used for conservative treatment. Clopidogrel hydrogen sulfate 75 mg po QD was given to the patient to inhibit platelet aggregation, pitavastatin was used to reduce blood lipid levels and stabilize plaques, butylphthalide was combined with edaravone dexborneol concentrated solution for injection, and urinary kallidinogenase injection to improve collateral circulation. The patient’s condition gradually stabilized. The physical examination revealed normal chest, clear respiratory sounds in both lungs, no dry and wet rales, no pleural frictional sound, rhythm, heart sound, and edema of both lower limbs. The physical examination of the nervous system showed that the patient was in good spirits, with no obvious abnormalities.

From the perspective of TCM, the patient’s disease belonged to the category of “heart failure,” combined with “cardiac distension,” “stroke,” “megrim,” and so forth. Referring to chronic heart failure integrated TCM and Western medicine diagnosis and treatment expert consensus,^[13]^ syndrome differentiation should be qi deficiency and blood stasis. Patient suffered from stagnant movement of Qi and blood stasis arised from the blood due to lack of yang qi in heart, Qi can control blood generation, distribution, and operation. The blood can carry and nourish qi. As the classic Chinese saying goes: qi is the commander of blood, and blood is the mother of qi. Blood-stasis toxin damages organs and causesyin and yang imbalance and qi deficiency. The movement of water in the body was impaired, with long accumulation and overflow in muscles. Moisture has the property of running downward. Therefore, edema was seen in the lower extremities, combined with the patient’s red tongue, thin tongue coating, pulse, and other manifestations, which belong to qi deficiency and blood stasis. In TCM treatment, the methods of invigorating the heart, promoting blood circulation, and removing blood stasis were as follows: Radix Astragali 45 g, Poria 30 g, Radix Codonopsis 30 g, and Polyporus 30 g applied to nourish the heart andinvigorate vital energy; and Pheretima 12 g, Rhizoma Curcumae 30 g, Radix Angelicae Sinensis 18 g, and Rhizoma Chuanxiong 18g applied to promote blood circulation and remove blood stasis, 1 dose daily, decocted 200 mL in water and taken warm in the morning and evening. After 3 doses, the symptoms of chest tightness were better than before, the tongue was light red, and the tongue was thin and white, but the patient still felt a slight difference in spirit, occasional fatigue, and sluggish pulse. This was the result of long-time illness, consumption of qi, and injury of the essence. Excessive anxiety led to stagnation of the circulation of vital energy. Therefore, 12 g Fructus Toosendan was added to the original prescription to relieve liver depression, and 30 g Rhizoma Cibotii to nourish the kidney and benefit the essence. The symptoms improved significantly, but the patients had edema of the lower limbs repeatedly, combined with a weak pulse. Hence, the edema syndrome due to kidney yang deficiency was considered. Therefore, on the basis of the second clinical prescription, Poria was discontinued. Tuckahoe Peel 30 g and Caulis Piperis Kadsurae 30 g were given for inducing diuresis and reducing edema, and Radix Dipsaci 30 g to improve the kidney. The patient still had edema and occasional lumbar weakness. The kidney yang deficiency syndrome was still treated according to syndrome differentiation, and Rhizoma Cibotii was discontinued according to the third prescription during treatment. Further, 30 g Herba Lophatheri was added for inducing diuresis and reducing edema, 30 g Cortex Eucommiae for enriching the kidney and strengthening yang-qi, and 12 g Cortex Phellodendri and 18 g Rhizoma Anemarrhenae for nourishing the kidney yin and reinforcing yang from yin. After 3 doses, the symptoms of chest tightness and suffocation significantly improved, with no edema in the lower limbs and no obvious discomfort.

**Figure 2.**
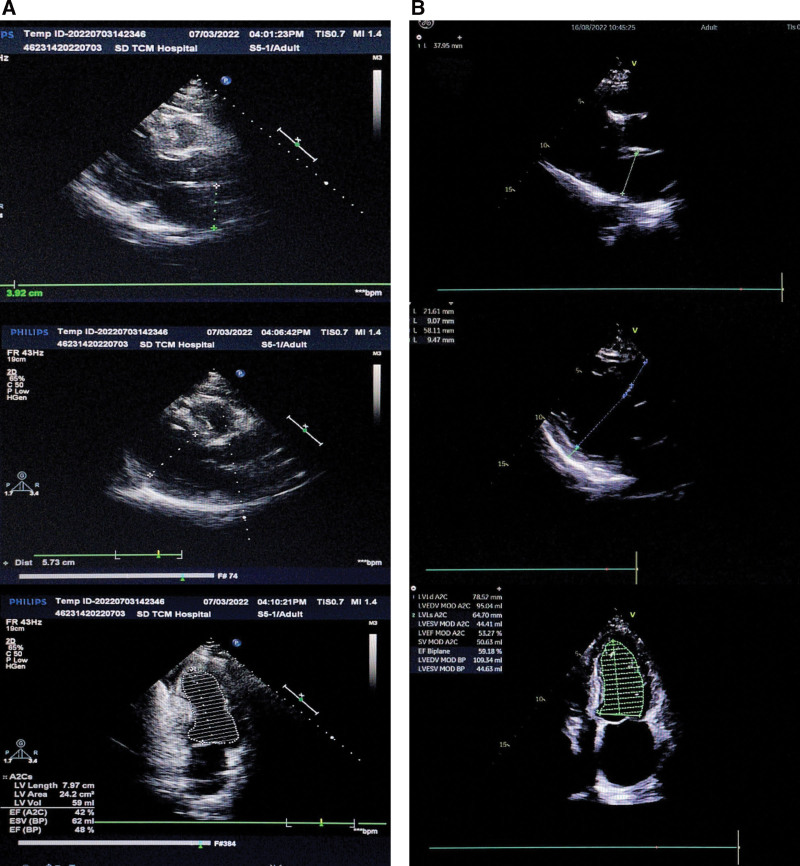
(A) Cardiac ultrasound within 2 mo outside the hospital. (a) Cardiac ultrasound on July 03, 2022: myocardial lesions; left ventricle slightly larger LAED 39 mm, LVED 57 mm); mitral regurgitation (mild); tricuspid regurgitation (mild); and reduced left ventricular systolic function (LVEF 0.48). (B) Cardiac ultrasound on August 16, 2022: left ventricle slightly larger (LAED 38 mm, LVED 58 mm); mitral regurgitation (mild); tricuspid regurgitation (moderate); and pulmonary hypertension; (LVEF 0.59). LAED = left atrium end diastolic, LVED = left ventricular end diastolic diameter, LVEF = left ventricular ejection fraction.

The patient’s cardiac function gradually recovered when out of the hospital (Fig. 2). The patient was regularly followed up in the outpatient department of cardiology of our hospital and prescribed rivaroxaban, spironolactone, clopidogrel hydrogen sulfate, sacubitril valsartan sodium, sodium bicarbonate, ivabradine hydrochloride, metoprolol succinate sustained-release tablet, pivastatin calcium, ezetimibe, and butylphthalide soft capsule (Table 1).

**Table 1:** Related adjustment of drug dose and type during treatment.

	Rivaroxaban	Spironolactone	Clopidogrel hydrogen sulfate	Sacubitril valsartan sodium	Sodium bicarbonate	Ivabradine hydrochloride	Metoprolol succinate sustained-release tablet	Pivastatin calcium	Ezetimibe	Butylphthalide soft capsule
June 15, 2022 to June 30, 2022	2.5 mg po BID	20 mg po QD	75 mg po QD	50 mg po BID	1 g po TID	5 mg po BID	23.5 mg po QD	2 mg po QN	10 mg po QD	200 mg po TID
June 30, 2022 to July 14, 2022	2.5 mg po BID	20 mg po QD	75 mg po QD	50 mg po BID	1 g po TID	5 mg po BID	23.5 mg po QD	2 mg po QN	10 mg po QD	200 mg po TID
July 14, 2022 to July 30, 2022	2.5 mg po BID	20 mg po QD	7 5 mg po QD	50 mg po BID	1 g po TID	Stop	23.5 mg po QD	Stop	10 mg po QD	200 mg po TID
July 30, 2022 to August 15, 2022	2.5 mg po BID	20 mg po QD	75 mg po QD	50 mg po BID	1g po TID	Stop	23.5 mg po QD	Stop	10 mg po QD	200 mg po TID
August 15, 2022 to September 01, 2022	2.5 mg po BID	20 mg po QD	75 mg po QD	50 mg po BID	0.5g po TID	Stop	47.5 mg po QD	Stop	10 mg po QD	200 mg po TID

**Figure 3.**
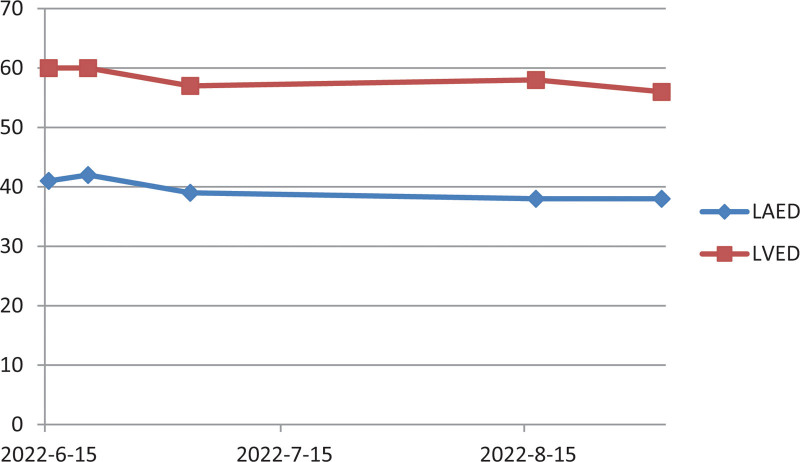
Changes in LVED and LAED during medication. LAED = left atrium end diastolic, LVED = left ventricular end diastolic diameter.

**Figure 4.**
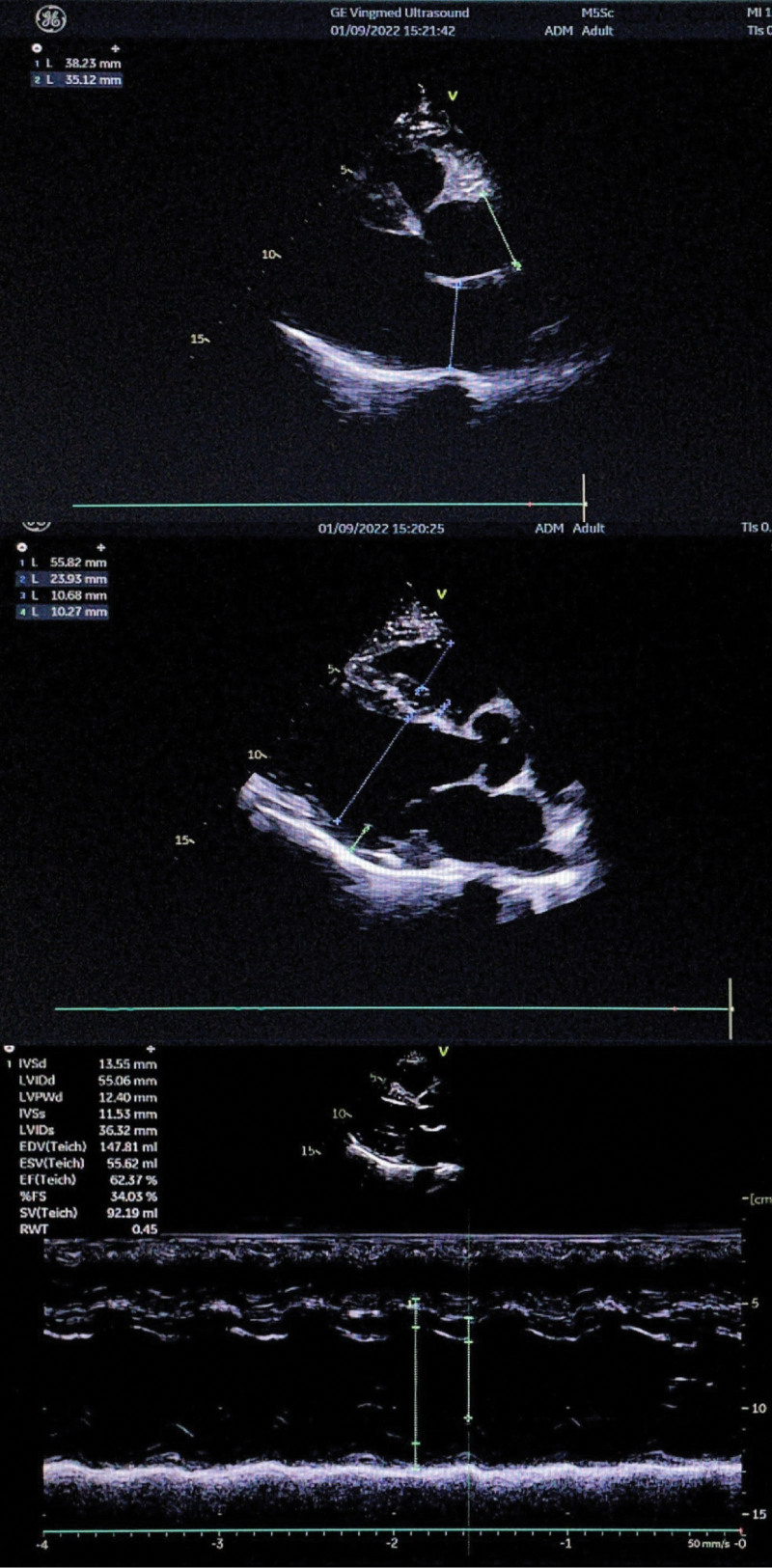
Recent follow-up results.

One month after the onset of acute cerebral infarction and acute heart failure, the patient’s condition gradually stabilized, DCM improved (Fig. 3), moyamoya disease was controlled, and heart-related indicators gradually recovered. On September 1, 2022, the cardiac function index was reexamined. The cardiac ultrasound indicated the following: left atrium end diastolic 38 mm, LVED 56 mm; and LVEF 62% (Fig. 4). He had no stroke or recurrence of heart failure.

## 3. Discussion

DCM has no obvious symptoms in the early stage. In the middle and later stages, it can easily develop into heart failure, ventricular or supraventricular arrhythmias, thromboembolism, sudden death, and so forth. Moyamoya disease mainly presents as intracranial hemorrhage or cerebral hemorrhage. This was a case report of DCM complicated with moyamoya disease. Reports on DCM with moyamoya disease are limited. The clinical evidence that DCM is significantly associated with moyamoya disease is still lacking. However, it is worth mentioning that in recent years, several studies have reported primary DCM^[14,15]^ with idiopathic moyamoya disease.^[16,17]^ The genetic studies on pathogenesis found that secondary DCM and moyamoya disease were mostly related to autoimmune disease and infection. They were associated with the inflammatory infiltration of cardiomyocytes and vascular endothelial cells.^[18]^ However, sufficient evidence on the correlation between DCM and moyamoya disease is still lacking, and hence further clinical and basic studies are needed.

According to the Cardiovascular Society of the Chinese Medical Association, among 10,714 patients with heart failure in 42 hospitals in China in 1980, 1990, and 2000, the proportion of DCM was 6.4%, 7.4%, and 7.6%, respectively. According to the Chinese Heart Failure Registry Study, DCM accounted for about 16%^[19]^ of 13,687 discharged patients with heart failure from 132 hospitals between 2012 and 2015. The prevalence of DCM increased every year. Enlarged heart late may result in arrhythmia, heart failure, thrombosis, and even death. The case fatality rate is higher. The cerebral examination of patients with DCM diagnosis showed moyamoya disease. Acute cerebral infarction symptom was more apparent. However, careful analysis of the primary disease revealed DCM with moyamoya disease. Therefore, the primary disease should be actively treated, correcting heart failure and improving cerebral infarction. The patients still need to use anticoagulation, thrombolysis, antiplatelet aggregation, and diuretic therapy. The treatment process has many contradictions and requires a balance between diseases, between drugs, and between diseases and drugs. Close attention should be paid to the changes in patients’ conditions, the cause should be analyzed accurately, and they should be better targeted for treatment.

## 4. Conclusion

We showed a case who had been diagnosed with DCM and moyamoya disease who demonstrated heart failure and acute cerebral infarction on the admission. Such findings might provide important information on the possibility of poor myocardial systolic function and abnormal cerebrovascular as the underlying cause of DCM and moyamoya disease in some patients.

## Author contributions

**Conceptualization:** Dong Xia Yun, Jie Yang, Chuan Hua Yang.

**Writing – original draft:** Dong Xia Yun, Jie Yang, Chuan Hua Yang.
